# Phenotypic plasticity and local adaptation favor range expansion of a Neotropical palm

**DOI:** 10.1002/ece3.4248

**Published:** 2018-07-03

**Authors:** Pedro H. S. Brancalion, Giancarlo C. X. Oliveira, Maria I. Zucchi, Mariana Novello, Juliano van Melis, Silvio S. Zocchi, Robin L. Chazdon, Ricardo R. Rodrigues

**Affiliations:** ^1^ Department of Forest Sciences “Luiz de Queiroz” College of Agriculture University of São Paulo Piracicaba SP Brazil; ^2^ Department of Genetics, “Luiz de Queiroz” College of Agriculture University of São Paulo Piracicaba SP Brazil; ^3^ Agribusiness Technological Development of São Paulo (APTA) Piracicaba SP Brazil; ^4^ Departament of Genetics and Molecular Biology Institute of Biology University of Campinas Campinas SP Brazil; ^5^ Department of Math, Chemistry and Statistics “Luiz de Queiroz” College of Agriculture University of São Paulo Piracicaba SP Brazil; ^6^ Department of Ecology and Evolutionary Biology University of Connecticut Storrs CT USA; ^7^ International Institute for Sustainability Rio de Janeiro RJ Brazil; ^8^ Department of Biology, “Luiz de Queiroz” College of Agriculture University of São Paulo Piracicaba SP Brazil

**Keywords:** Atlantic Forest, common garden, ecological ranges, ecotypes, *Euterpe edulis*, evolutionary ecology, next‐generation sequencing, reciprocal transplants, SNP genotyping

## Abstract

One of the most intriguing questions in plant ecology is which evolutionary strategy allows widely distributed species to increase their ecological range and grow in changing environmental conditions. Phenotypic plasticity and local adaptations are major processes governing species range margins, but little is known about their relative contribution for tree species distribution in tropical forest regions. We investigated the relative role of phenotypic plasticity and local adaptation in the ecological distribution of the widespread palm *Euterpe edulis* in the Brazilian Atlantic Forest. Genetic sampling and experiments were performed in old‐growth remnants of two forest types with higher (Seasonal Semideciduous Forests vs. Submontane Rainforest) and lower biogeographic association and environmental similarities (Submontane Rainforest vs. *Restinga* Forest). We first assessed the molecular genetic differentiation among populations, focusing on the group of *loci* potentially under selection in each forest, using single‐nucleotide polymorphism (SNPs) outliers. Further, we looked for potential adaptive divergence among populations in a common garden experiment and in reciprocal transplants for two plant development phases: seedling establishment and sapling growth. Analysis with outlier loci indicated that all individuals from the Semideciduous Forest formed a single group, while another group was formed by overlapping individuals from Submontane Rainforest and *Restinga* Forest. Molecular differentiation was corroborated by reciprocal transplants, which yielded strong evidence of local adaptations for seedling establishment in the biogeographically divergent Rainforest and Semideciduous Forest, but not for *Restinga* Forest and Submontane Rainforest. Phenotypic plasticity for palm seedling establishment favors range expansion to biogeographically related or recently colonized forest types, while persistence in the newly colonized ecosystem may be favored by local adaptations if climatic conditions diverge over time, reducing gene flow between populations. SNPs obtained by next‐generation sequencing can help exploring adaptive genetic variation in tropical trees, which impose several challenges to the use of reciprocal transplants.

## 1 INTRODUCTION

Tropical forests are the most biologically diverse ecosystems on the Earth, with an estimated 53,000 tree species (Slik et al., 2015). This high diversity is associated with high levels of endemism (Hubbell, 2013; Myers et al., 2013). The spatial patterns of plants in tropical forests are created by a combination of environmental gradients, ecological drift, dispersal limitations, barriers allowing vicariance and dispersal events, and stable climate, which drive evolutionary processes over millions of years (Chave, 2004; Davis, Shaw, & Etterson, 2005; Dexter, Terborgh, & Cunningham, 2012; Schemske, 2002; Usinowicz et al., 2017). A narrow range distribution is typically accompanied by habitat specialization in tropical trees, in response to factors such as local environmental conditions, ecological interactions (Fine, Mesones, & Coley, 2004), and local resource availability (Condit, Engelbrecht, Pino, Perez, & Turner, 2013; Palmiotto et al., 2004; Svenning, 1999). Despite high levels of endemism in tropical forests, many species have a wide geographic distribution and can grow and reproduce in habitats with contrasting ecological conditions (Dick & Heuertz, 2008; Pennington & Lavin, 2016).

Understanding the evolutionary strategy that allows widely distributed species to increase their ecological range and grow in diverging environmental conditions is a foundational question in plant ecology (Sutherland et al., 2013). Initially, the geographic spread of species is governed by dispersal mechanisms and is favored by high phenotypic plasticity (Sexton, McIntyre, Angert, & Rice, 2009). Phenotypic plasticity implies “the ability of an individual organism to alter its physiology/morphology in response to changes in environmental conditions” (Schlichting & Levin, 1986). Phenotypic plasticity can occur during a single generation and does not require genetic change in an individual or in a population. But persistence in the newly colonized region may require further genetic differentiation if ecological or climatic conditions diverge (Ronquist, 1997). Local adaptation—the average higher fitness, resulted from divergent natural selection, of local genotypes compared to genotypes from other habitats (Kawecki & Ebert, 2004)—may thus become a key evolutionary process to consolidate species range expansion and, further, speciation, if sufficient genetic divergence occurs.

Plasticity and adaptation are highly intertwined processes. Phenotypic plasticity can be adaptive or maladaptive (Pigliucci, Murren, & Schlichting, 2006), as high plasticity can entail a cost in growth and survival (Hendry, 2016). As it is not likely that the populations are genetically preadapted to newly encountered environmental conditions, a sufficiently broad range of plasticity allows them to survive in the new habitats. Eventually, after allopatric populations have grown for successive generations in their new habitats, the increase in genetic divergence among them in response to disruptive selection may generate ecotypes (Hufford & Mazer, 2003; Linhart & Grant, 1996). Both local adaptation and phenotypic plasticity—and their interaction—are likely to occur at the same time in natural populations, although the contribution of these processes for range expansion is not yet fully understood, especially for long‐lived plants (Valladares et al., 2014).

The relative contributions of plasticity and adaptation in governing species range margins are expected to depend on the biogeographic context. Phenotypic plasticity could be sufficient to support persistence of species in adjacent biogeographic regions despite their different edaphic characteristics, whereas genetic adaptation is potentially needed, in addition to phenotypic plasticity, for persistence of populations in distinct climate zones and distant biogeographic regions (Valladares et al., 2014). Although local adaptation could also increase fitness in adjacent biogeographic regions, high gene flow would prevent genetic differentiation among population, so phenotypic plasticity may remain as the major evolutionary driver of differential population fitness in different habitats.

In this context, we investigated the roles of phenotypic plasticity and local adaptation in the ecological distribution of a Neotropical, threatened, and once widely distributed palm in different forest types of the Brazilian Atlantic Forest, one of the most species‐rich and threatened ecosystems on the Earth (Joly, Metzger, & Tabarelli, 2014). We explored the range distribution of the economically and ecologically important palm species *Euterpe edulis* in three forest types within the Atlantic Forest biome. We compared phenotypic plasticity and local adaptation of *E. edulis* populations (a) between major forest types submitted to historical biogeographic distinction (Semideciduous Forest in a seasonal climate, nutrient‐rich soils, and distributed in inland regions vs. Submontane Rainforest in wet climate, nutrient‐poor soils, and distributed along coastal regions) and (b) within a major forest type (Rainforest) submitted to shorter‐term biogeographic distinction, as a consequence of the recent colonization of the younger ecosystem by the species of its neighboring ecosystem (Submontane Rainforest vs. *Restinga* Forest in sandy coastal plains at the margins of Submontane Rainforests). *E. edulis* is the only species of this genus found in the Atlantic Forest, out of the center of endemism of *Euterpe* in the Amazon. Invasion of the *E. edulis* ancestor into the Atlantic Forest may have occurred in northeastern Brazil during the Pliocene and Pleistocene, when the climate was more humid and the Atlantic Forest was connected to the Amazon in this region (Reis, Guerra, Nodari, Ribeiro, & Reis, 2000). Once *E. edulis* evolved from its invading ancestor and established in both inland Semideciduous Forests and coastal Rainforests, cyclical changes in climate may have reduced the connection between these ecosystems through the expansion of intervening savannahs and intensified the ecological divergences among them, thus creating favorable conditions for genetic isolation and local adaptations (different climate and soil; reduced dispersal). The invasion of *Restinga* Forests by *E. edulis* individuals coming from neighboring Submontane Rainforests is a contemporary and continuous process that may increase gene flow, as Lowland and Submontane Rainforests have always been connected to *Restinga* Forests (similar climate, different soils, and high dispersal; Marques, Swaine, & Liebsch, 2011).

The high adaptive potential of *E. edulis* may have contributed to its wide ecological and geographic distribution in the Atlantic Forest biome. In fact, morphological varieties of *E. edulis* have been found across the Atlantic Forest, including a variety with multiple stems (Portela, Pires, & Santos, 2009). One of the varieties with a yellow leaf sheath was once classified as a new species (*E. espiritosantensis* H.Q.B. Fernandez; Wendt et al., 2011), but molecular analysis with isoenzymes did not find relevant differentiation (Martins et al., 2007), and *E. espiritosantensis* is no longer considered a valid species. Populations with smaller seeds are found in defaunated forests, as a consequence of a rapid evolutionary change driven by the ecological extinction of large‐gaped birds (Carvalho, Ribeiro, Cortes, Galetti, & Collevatti, 2015; Galetti et al., 2013). Molecular analyses have corroborated the explanations of population divergences according to the site of origin (Gaiotto, Grattapaglia, & Vencovsky, 2003) and to forest fragmentation levels (Carvalho et al., 2015), but neither the investigation of adaptive molecular divergence with SNPs, nor reciprocal transplant experiments, had been performed for *E. edulis* so far. In addition, investigating the adaptive potential of *E. edulis* may be critical to assess whether this threatened species will be able to overcome the selective barriers imposed by climate change—a major threat for plant species conservation in the Atlantic Forest (Colombo & Joly, 2010)—as reduced population size and gene flow caused by habitat fragmentation and overexploitation of palm heart, summed with the vulnerability of *E. edulis* to drought (Silva‐Matos & Alves, 2008), may compromise the persistence of this species under a changing climate.

Our main hypothesis is that phenotypic plasticity is sufficient for expanding *E. edulis* range to more biogeographically similar ecosystems and that local adaptations are required, in addition to phenotypic plasticity, to allow its occurrence in habitats with higher biogeographic distinction. Under local adaptation, we expected enhanced seedling establishment (i.e., higher seedling emergence, survival, growth, and density) and sapling development (i.e., higher biomass, survival, and root–shoot ratio in drier ecosystems) for each local provenance in each site, while we expected phenotypic plasticity to lead to among‐site variations in the aforementioned traits, regardless of the superior plant performance of local genotypes.

To test the aforementioned predictions, we combined two methods traditionally employed in evolutionary studies (common garden and reciprocal transplants) with a novel molecular approach to estimate the adaptive differentiation among populations, using single‐nucleotide polymorphisms (SNPs). The use of these traditional methods for a tropical palm in different forest types, integrated into SNP analysis, may further contribute to advance the investigation methods of phenotypic plasticity and adaptation in tropical forests. Reciprocal transplants have predominantly been applied for herbaceous and shrubby species growing in temperate regions (Fenster & Galloway, 2000; Franks, Weber, & Aitken, 2014; Grassein, Lavorel, & Till‐Bottraud, 2014; Malikova, Latzel, Smilauer, & Klimesova, 2016), with few exceptions in the tropics (Chen & Schemske, 2015), because it may take several years to evaluate the overall plant performance during an individual's life span. Although all the phases should be studied in order to obtain the complete picture of ecotypic adaptations, in arboreal species it may be methodologically appropriate, if not plainly inevitable, to split the cycle into practical sets of phases: As Darwin 1994, page 86, put it: “… so in a state of nature, natural selection will be enabled to act on and modify organic beings at any age, by the accumulation of profitable variations at that age, and by their inheritance at a corresponding age.” As significant plant mortality occurs during the seedling establishment and sapling growth phenophases, natural selection should in theory strongly benefit variants within plant populations with higher plant performance in these phases (Postma & Agren, 2016). Hence, plant performance during seedling establishment and sapling initial growth of long‐lived species should logically be a reliable indicator of ecotypic differentiation in the context of range size and habitat distribution. However, establishing reciprocal transplant experiments will always be a practical challenge for studying adaptation in the thousands of tropical plant species.

Novel genomic methods may further help overcoming the aforementioned barriers for studying adaptive variation in species‐rich communities dominated by long‐lived plants (Allendorf, Hohenlohe, & Luikart, 2010). Next‐generation sequencing allows the discovery of single‐nucleotide polymorphisms (SNPs) and the identification of candidate *loci* (outliers) involved in local adaptation, with reduced cost even in nonmodel species (Allendorf et al., 2010; De Kort et al., 2014; Funk, McKay, Hohenlohe, & Allendorf, 2012). Different approaches have been developed for detecting diversifying selection. Considering that divergent natural selection is expected to increase levels of differentiation between populations, F_ST_ outlier approaches, based on levels of differentiation between populations, are being widely used. These were developed to test whether the level of differentiation at a particular locus is excessively high compared to the expectation of neutral variation (Bragg, Potter, Bi, & Moritz, 2016; Bragg, Supple, Andrew, & Borevitz, 2015; Storz, 2005; Storz & Hoekstra, 2007). The integration of reciprocal transplants with next‐generation sequencing could yield substantial advances in evolutionary biology and conservation genetics, by identifying key genome regions under selection in different ecological contexts and the immediate consequences of molecular differentiation on adaptive traits (Barrett & Hoekstra, 2011; De Kort et al., 2014). In this study, we use an integrated approach of a common garden, reciprocal transplants, and SNP genotyping, to investigate the interplay between phenotypic plasticity, local adaptation, and ecological distribution of populations of the palm *E. edulis*.

## 2 MATERIAL AND METHODS

### 2.1 Study sites

We conducted our study in the Brazilian Atlantic Forest, a top five global hotspot for biodiversity conservation due to its high levels of biodiversity and endemism associated with a dramatic threatened status (Myers, Mittermeier, Mittermeier, da Fonseca, & Kent, 2000). This was the first tropical forest that Charles Darwin had ever visited, as commented in a letter to J. S. Henslow sent in 1832: “Here I first saw a tropical forest in all its sublime grandeur.—Nothing, but the reality can give any idea, how wonderful, how magnificent the scene is” (Darwin, 1832). Only 12% forest cover remains, distributed as small and isolated remnants, over eight main biogeographic subregions (Ribeiro, Metzger, Martensen, Ponzoni, & Hirota, 2009). The dominant forest types are the Seasonal Semideciduous Forest (hereafter Semideciduous Forest) and the Dense Ombrophilous Forest (Morellato & Haddad, 2000). Semideciduous Forests occur predominantly in inland regions, on nutrient‐rich soils, and in seasonal climates, with a moderate dry season and reduced rainfall, while Dense Ombrophilous Forests are distributed along the Atlantic coast, on nutrient‐poor soils and rainy climates without a biologically dry season. The substantial floristic differentiation of tree species between these forest types is strongly driven by rainfall and seasonality (Eisenlohr & de Oliveira, 2015; Oliveira & Fontes, 2000).

Dense Ombrophilous Forests show distinct ecological features and classification according to the altitudinal range where they occur: Lowland: 0–50 m asl; Submontane: 50–500 m asl; and Montane: 500–1,500 m asl. In fact, these rainforest subtypes diverge in floristic composition, which is strongly associated with variation in elevation and associated temperatures (Oliveira & Fontes, 2000). We studied here a Submontane Dense Ombrophilous Forest (hereafter Rainforest) and a particular type of Lowland Dense Ombrophilous Forest, locally known as *Restinga* Forest, which is distributed over geologically young soils formed by sandy deposits created and destroyed by cyclical changes in the sea level from the Pleistocene to the Holocene (Marques et al., 2011). Given the particular features of *Restinga* Forests (swampy and sandy soils, with high sodium content and strong influence of sea‐level variation; (Brancalion, Vidal, Lavorenti, Batista, & Rodrigues, 2012), compared to regular Lowland Rainforests (better drained and clay soils with regular sodium content and no influence of sea‐level variation), we will follow the recommendation of Marques et al. (2011) and hereafter refer to this subtype of Lowland Rainforest as *Restinga* Forests. *Restinga* Forests lack endemic woody species and are marginal habitats predominantly formed by repeated colonization events from neighboring Rainforests distributed across the Atlantic mountain range (Marques et al., 2011; Scarano, 2002).

We conducted our study in some of the last well‐conserved remnants of these three forest types: Caetetus Ecological Station (Semideciduous Forest; 2,254 ha), Carlos Botelho State Park (Rainforest; 37,644 ha), and Ilha do Cardoso State Park (*Restinga* Forest; 13,500 ha), all of them located in the state of São Paulo, southeastern Brazil (Supporting information: Figure S1). A detailed description of the environmental conditions of these sites is provided in Supporting information: Table S1.

### 2.2 Study species

We selected a palm species for this study because of the particular evolutionary history of Arecaceae, through which their modern geographic patterns of diversity have been associated with the combination of dispersal and vicariance events (Bjorholm, Svenning, Skov, & Balslev, 2005). In this context, the huge ecological heterogeneity of tropical ecosystems allowed Arecaceae to constitute one of the most diversified plant families in the tropics (Eiserhardt, Svenning, Kissling, & Balslev, 2011) and offer an attractive model for evolutionary studies. We chose the palm species *Euterpe edulis* Mart., one of the 66 total and 44 endemic palm species growing in the Atlantic Forest of Brazil (REFLORA, 2017). This is the only *Euterpe* species in Brazil occurring outside the Amazon region, the center of origin of this genus of 30 species. It is a single‐stemmed, 5‐ to 15‐m‐tall palm, widely dispersed within the biome, between latitudes 15°S and 29°S (Cardoso, Eloy, Provan, Cardoso, & Ferreira, 2000). It was the most abundant species in the study areas of Rainforest and *Restinga* Forest, corresponding, respectively, to 19.8 percent and 21.5 percent of the stems with diameter at breast height >4,8 cm (Brancalion et al., 2012). Such widespread and abundant species is especially prone to geographic diversification, and molecular differentiation among *E. edulis* populations was found to be closely related to spatial proximity (Cardoso et al., 2000). *Euterpe edulis* has been considered a keystone species for frugivores in the Atlantic Forest, as it produces fruits that are consumed and dispersed by at least 30 bird and 15 terrestrial mammal and bat species. Illegal harvesting of this palm has driven the decline of the species, and it is now classified as threatened with extinction in Brazil (Muler et al., 2014). It produces globular, recalcitrant seeds (*ca*. 45% seed moisture content) of ~10 mm diameter and 0.6 g of dry weight (Brancalion, Novembre, & Rodrigues, 2011). More details on its reproductive biology and effects of environmental conditions in plant growth and seed production can be found in Brancalion et al. (2012).

### 2.3 Genetic sampling and analysis

We sampled roots from adult individuals (Semideciduous Forest: *n *=* *19; Rainforest: *n *=* *20; and *Restinga* Forest: *n *=* *16) at least 100 m apart from each other. The samples were stored in liquid nitrogen in the field and further transferred to a −80°C freezer in the laboratory. Genomic DNA was extracted from root tissues using a standard cetyltrimethyl ammonium bromide protocol (Doyle & Doyle, 1987). Double‐digest restriction‐associated DNA (ddRAD) libraries were prepared using about 150 ng of DNA per sample following the protocol described by Peterson (Peterson, Weber, Kay, Fisher, & Hoekstra, 2012). A ddRAD library was prepared with all samples and further diluted and sequenced in two lanes of a NextSeq 500 (Illumina, Inc.). Sequencing data were analyzed in STACKS platform (Catchen, Hohenlohe, Bassham, Amores, & Cresko, 2013).

We used the program *ustacks* for aligning the sequences and forming short‐read sequence stacks to enable the detection of SNP loci contained within the stacks; the parameters used in this process were minimum stack depth (*m* = 3) and maximum distance allowed between stacks to merge them into a locus (M = 2). With tags formed by *ustacks*, we built a catalog with *cstacks* to serve as a reference genome. Sets of stacks constructed by the *ustacks* program were matched against the catalog through the *sstacks* program. Afterward, the genotypes of the individuals were corrected with the *rxstacks* program, based on the data from populations. The *populations* package was used for filtering the dataset to obtain good‐quality SNPs for the calculation of population genomic statistics, following the criteria: minimum minor allele frequency (min_maf = 0.01); minimum stack depth of coverage required for each individual (*m* = 3); and minimum percentage of individuals in a population required to process a locus for that population (*r* = 0.6).

The population genomic diversity of populations was estimated according to the expected (*H*_E_) and observed heterozygosity (*H*_O_), using the PopGeneKit package (Paquete, 2012), and the estimated number of alleles (*A*), allelic richness (*A*_R_), and inbreeding coefficients (*F*_is_), using the R package diversity (Keenan, McGinnity, Cross, Crozier, & Prodohl, 2013). Both packages were written in R (R Development Core Team, 2016). Genetic differentiation was estimated through the overall and pairwise *F*_ST_ values (Weir & Cockerham, 1984), using the R package *diversity* (Keenan et al., 2013). Population structure was inferred using the discriminant analysis of principal components (DAPC) (Jombart, Devillard, & Balloux, 2010), a multivariate analysis to evaluate the genetic population structure, using the R package *adegenet* (Jombart, 2008). The hierarchical distribution of the genomic variation between and within populations was assessed through an analysis of molecular variance (AMOVA) (Michalakis & Excoffier, 1996), using the *poppr* package in R (Kamvar, Tabima, & Grunwald, 2014). The significant level was obtained based on 10,000 permutations.

Outlier loci were assessed based on the relationship between F_ST_ and expected heterozygosity (*H*_E_) according to an island model with neutral markers (Beaumont & Nichols, 1996), using the program LOSITAN (Antao, Lopes, Lopes, Beja‐Pereira, & Luikart, 2008). This method assumes that loci with *F*_ST_ excessively high or low, compared to the expectations under neutrality conditions, are candidates to be under selection (Antao et al., 2008; Schoville et al., 2012). LOSITAN was run in pairs, considering three scenarios: Semideciduous Forest versus Rainforest, Semideciduous Forest versus *Restinga* Forest, and Rainforest versus *Restinga* Forest, using the parameter setting of 100,000 simulations, confident interval of 0.95, and false discovery rate (FDR) of 0.1. Outlier SNPs were used in nucleotide searches with BLASTx against the genomic database of the National Center for Biotechnology Information, using blast2go (Gotz et al., 2008). For the sequences with significant BLAST hits, functional annotation was taken using the ontology system “gene ontology.”

### 2.4 Seed collection

Seed harvesting was carried out concomitantly in each study site, within the same populations and using approximately half of the individuals sampled for molecular analysis. Five mother palms, at least 100 m apart, were sampled from two distinct parts of each protected area (total of 10 mother palms per site). We used the same seed lots, obtained from the same mother palms in a single harvesting event, for all the experiments.

### 2.5 Common garden experiment

We assessed the growth potential and biomass allocation of seedlings harvested in the three aforementioned populations under favorable, common garden conditions, established in a forest nursery based on the recommended guidelines for commercial *E. edulis* seedling production. Two saplings produced with the seeds from each mother palm (total of 30 mother palms), thus reproducing the sapling design of seed collection, were grown in plastic pots (22 cm height × 13 cm diameter) containing fertilized soil, which were randomly distributed and kept in forest nursery conditions (50% shading and three daily irrigations (Brancalion, Rodrigues, & Oliveira, 2015). The experiments were evaluated according to the dry mass of roots, leaves, and shoot, total dry mass, and root–shoot dry mass ratio of 12‐month‐old seedlings (oven‐dried at 72°C for 48 hr).

### 2.6 Reciprocal transplants using seeds

Reciprocal transplants using seeds were implemented 7 days after seed harvesting to avoid the viability loss of the recalcitrant seeds of *E. edulis* (Brancalion et al., 2011). Eight experimental blocks were set up along two different trails in each protected area, each block containing three plots randomly distributed with 30 seeds per provenance (three seeds for each mother palm and ten mother palms per forest type). As every mother palm supplied seeds for all experiments, at least half of their genome was exactly repeated in the three forest types. The experimental blocks were protected by exclusion cages (110 cm long × 70 cm wide × 15 cm high) made with iron rods and covered by a wire mesh with round 1‐cm‐diameter openings to avoid the seed removal by scatter‐hoarding mammals and seed deposition by dispersal agents. Seeds were buried in the ground at a depth equivalent to one half of their diameter, and the other half was left exposed. We evaluated the number of seedlings present in the plots 90, 180, and 270 days after sowing, when complete endosperm exhaustion was observed. Seedlings were individually marked across the monitoring period with plastic tags in order to evaluate the emergence and mortality (Supporting information: Figure S1). Seedling establishment performance was evaluated according to the (a) seedling emergence, (b) seedling mortality, (c) final seedling density, and (d) aboveground dry mass per seedling. We are aware that except for “final seedling density,” these performance traits may not provide a direct evidence of local adaptation, as other potential effects (e.g., maternal effects and soil pathogen communities) may have led to patterns of local provenances outperforming the foreign provenance at one site (Kawecki & Ebert, 2004). In spite of these limitations, these performance traits may have a close link with local adaptation due to the association between survival and growth with the reproduction potential of individuals; further, evaluating the adaptive traits traditionally used to assess improved plant performance in plant evolutionary studies would not be viable for palm species, whose life cycle may prolong for decades.

### 2.7 Reciprocal transplants using seedlings

The seedlings used in this experiment were produced according to the same nursery conditions described in the common garden experiment, except for the substrate and recipient used; seedlings were grown in 56‐cm³ plastic tubes filled with a nutrient‐free organic growing mixture, to avoid any influence in seedling nutritional status that could mask the effect of local soil on their growth. When seedlings showed one full‐expanded primary leaf, they were transplanted in experimental plots established inside undisturbed regions of the protected area in the beginning of the rainy season, two meters apart from the same experimental blocks used in the reciprocal transplants using seeds. Ten seedlings from each seed provenance (one seedling representing each mother plant) were randomly planted in each plot 30 cm apart from each other (30 seedlings per plot). Sapling growth performance was evaluated according to the survival, total dry mass, and root–shoot dry mass ratio of 12‐month‐old saplings (oven‐dried at 72°C for 48 hr; Supporting information: Figure S1). The same limitations described above, on the use performance traits to evaluate the fitness in the reciprocal transplant experiment using seeds, apply here too.

### 2.8 Data analysis of common garden and reciprocal transplants

All statistical analyses and model selections were constructed by applying generalized or standard linear models (GLM or SLM) (Faraway, 2016; Zuur, Ieno, Walker, Saveliev, & Smith, 2009). When considering counting data (seedling emergence, mortality, and final density) as response variables, we used a Poisson's or binomial error distribution; otherwise, we used Gaussian error distribution for traits (seedling aboveground dry mass, sapling total dry mass, and shoot–root dry mass ratio), using log‐transformed data. Our full model for reciprocal transplants included maternal origin of seed, local of transplant (i.e., local of seed sowing or seedling plantings), and their interaction as independent variables. In order to reduce the bias, we first analyzed the influence of seed mass values on our models. Seed mass did not show a significant effect on counting variables (data not shown), so it was not included in these models. However, seed mass showed importance in the models for other variables and was therefore included in the models. To determine the best model, we used an information theoretical approach based on the Akaike information criterion (AIC), by which the best model was indicated by the AIC lower value under the condition that ΔAIC is no higher than 2. All best models were validated by exploring their residuals. Also, we performed Fisher's exact test for count data in order to check the null hypothesis of independence between observed values of count data and seed origin for each studied area. Finally, we performed paired analyses between plants with distinct maternal origin for each local of transplant. We used Mann–Whitney tests for counting data and t‐tests for the log‐transformed data. These analyses were performed in R version 3.3.1 (R Development Core Team 2016).

## 3 RESULTS

### 3.1 Molecular characterization and differentiation of populations

**Figure 1:**
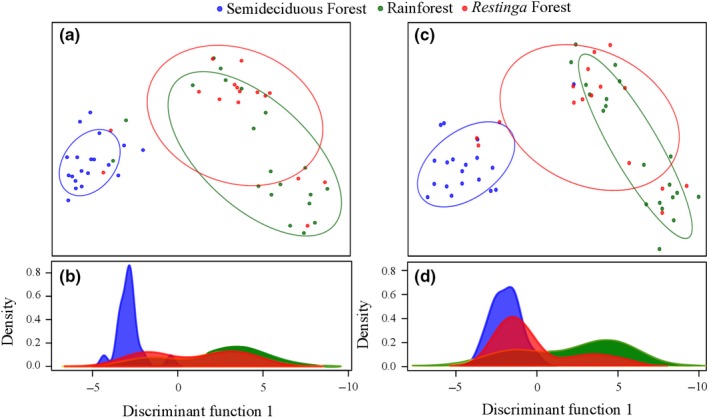
Genetic structuring using 501 neutral and 84 outlier SNPs of *Euterpe edulis* populations from different forest types of the Brazilian Atlantic Forest: scatter plot of clusters of individuals and density plots of individuals using neutral loci (a and b, respectively) and outlier loci (c and d, respectively)

A total of 808 SNPs were retained after the sequencing filter in STACKS for the 55 individuals analyzed. Analysis with LOSITAN in the three *E. edulis* populations detected 501 neutral SNPs that were used for genomic analysis and 307 outlier loci, with 84 of these possibly under divergent selection and 223 putatively under balancing selection. Overall, genomic diversity parameters were similar among populations, but average allelic richness was significantly higher in the Semideciduous Forest (Table 1). Most of the variation was attributed to intrapopulational diversity (global *F*_ST_ values of 0.064). We found a low level of genetic differentiation between Rainforest and *Restinga* Forest populations (pairwise *F*_ST_ = 0.01), and intermediate levels between these two populations and the Semideciduous Forest population (*F*_ST_ = 0.08; Table 2). In fact, the analysis of molecular variance (AMOVA) confirmed that most (82.72%) of the variation was found within populations (Table 3), but the genetic differentiation between populations and the forest types they represent was high and significant (θ = 0.173). Three distinct genetic groups were formed according to the DAPC analysis, based on Bayesian inference criterion (Figure 1). For neutral loci, all individuals from the Semideciduous Forest formed a single, but not exclusive, group, whereas another group was formed by overlapping individuals from Rainforest and *Restinga* Forest (Figure 1A,B); similar results were found when outlier loci were considered (Figure 1C,D).

**Table 1:** Genomic diversity parameters using 501 neutral SNPs of three *Euterpe edulis* populations from different forest types of the Brazilian Atlantic Forest

Forest types	*N*	*A*	*H*_o_	*H*_E_	Ar	*F*_IS_
Semideciduous Forest	19	897	0.149 (0.153–0.194)	0.129 (0.135–0.162)	1.58*	−0.1653
Rainforest	20	743	0.176 (0.137–0.184)	0.134 (0.113–0.142)	1.36	−0.2537
*Restinga* Forest	16	706	0.160 (0.129–0.175)	0.116 (0.101–0.131)	1.34	−0.3021

*Notes*

Number of individuals (*N*), mean number of alleles (*A*), observed heterozygosity (*H*_o_), expected heterozygosity (*H*_E_), allelic richness (Ar), and inbreeding coefficient (*F*_IS_). The values in parentheses correspond to the upper and lower limits of the confidence interval. * indicates significantly higher values.

**Table 2:** Population pairwise F_ST_ values (lower triangle) and 95% confidence intervals (upper triangle) for 501 neutral SNP loci of three *Euterpe edulis* populations from different forest types of the Brazilian Atlantic Forest (Global *F*_ST_ = 0.064)

Forest types	Semideciduous Forest	Rainforest	*Restinga* Forest
Semideciduous Forest	–	0.057–0.108	0.059–0.102
Submontane Rainforest	0.082	–	−0.001 to 0.023
*Restinga* Forest	0.080	0.011	‐

**Table 3:** Genetic differentiation within and among three *Euterpe edulis* populations from different forest types of the Brazilian Atlantic Forest according to analysis of molecular variance (AMOVA)

Source of variation	*df*	Sum of squares	Coefficient of variation	Percentage of variation (%)	θ statistics	*p*
Among populations	2	2197.3	47.6	17.27	θ = 0.173	<0.0001
Within populations	52	11875.1	228.3	82.72		

**Figure 2:**
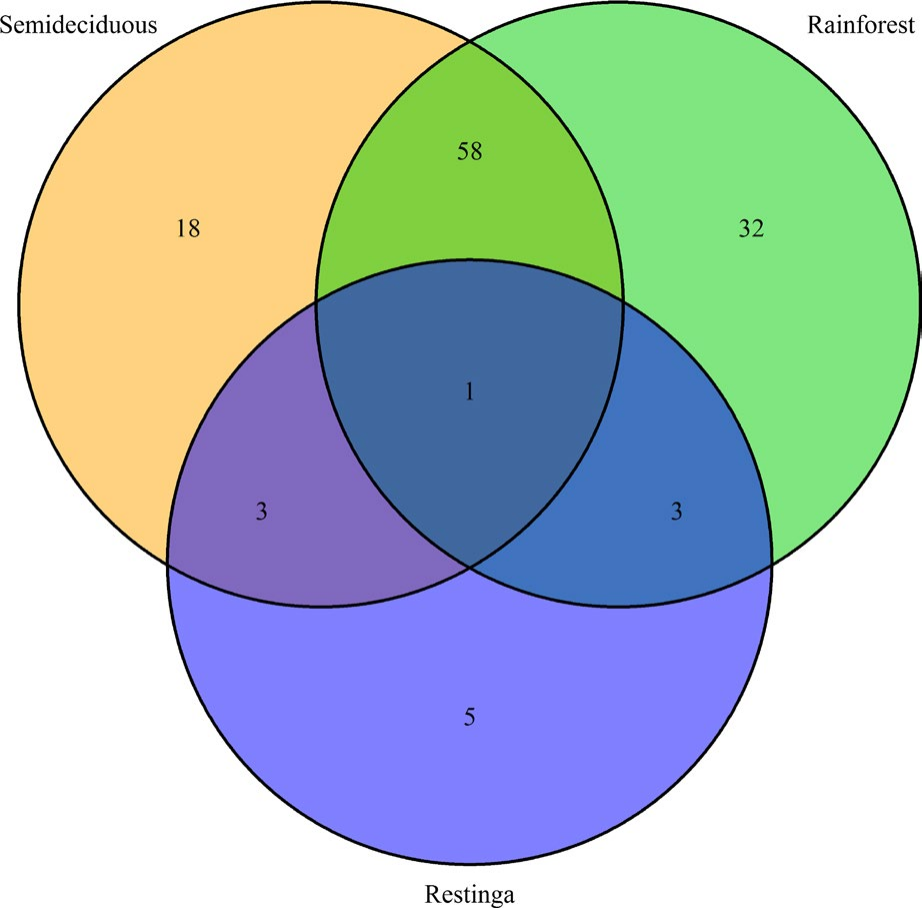
Venn diagram indicating the common outlier *loci* of *Euterpe edulis* among forest types of the Brazilian Atlantic Forest

A high number of outlier SNPs possibly under divergent selection were detected when the Semideciduous Forest was compared both with Rainforest (80) and with *Restinga* Forest (94), while only 12 were found for Rainforest versus *Restinga* Forest. The number of contigs with a significant number of blast hits in the GenBank database followed a similar pattern: 10 for Semideciduous Forest versus Rainforest, 12 for Semideciduous Forests versus *Restinga* forest, and only one for Rainforest versus *Restinga* Forest. Only one outlier locus was shared among all three populations (Figure 2). Gene ontology functional annotation of contigs was classified according to the GO term (Supporting information: Table S2). The gene ontology terms were assigned to different functional groups (cellular component, molecular function, and biological process), with three categories each (Supporting information: Figure S2). Within “molecular function,” the largest proportion of contigs was assigned to “binding” and “catalytic activity,” while for “biological process,” most contigs were involved in “cellular metabolic process” (Supporting information: Figure S2).

### 3.2 Common garden and reciprocal transplant experiments

We did not observe a significant influence of seed provenance on the root–shoot dry mass ratio (*F*_57,2_ = 1.50) and dry mass of roots (*F*_57,2_ = 2.42), shoot (*F*_57,2_ = 1.68), leaves (*F*_57,2_ = 1.33), and the whole individual (*F*_57,2_ = 1.95) of saplings grown in the common garden experiment (Exp. #1). However, substantial effects on seed provenance were observed in the reciprocal transplants using seeds and seedlings (Table 4).

**Table 4:** Selection of models according to the Akaike information criterion (AIC) for investigating the effect of seed provenance and site of transplant on seedling and sapling adaptive traits assessed in reciprocal transplants with *Euterpe edulis* in different forest types of the Brazilian Atlantic Forest

Forest types	Adaptive trait	ΔAIC				
		Seed provenance	Site of transplant	Both	With interaction^a^	Seed mass inclusion^b^
Semideciduous Forest versus Rainforest	Seedling emergence	100.53	28.26	19.30	**0.00**	
	Seedling mortality	267.34	5.39	1.19	**0.00**	
	Seedling density	33.88	39.37	35.77	**0.00**	
	Seedling aboveground dry mass	28.15	38.13	3.81	**0.00**	Yes
		49.20	53.21	24.91	22.31	No
	Sapling survival	**0.00**	1.00	1.91	0.21	
	Sapling root–shoot dry mass ratio	5.13	8.34	**0.00**	1.78	Yes
		5.79	8.45	0.45	2.18	No
	Sapling total dry mass	7.71	**0.00**	1.59	3.57	Yes
		11.57	3.54	5.21	7.20	No
Rainforest versus Restinga Forest	Seedling emergence	**0.00**	3.17	1.27	3.06	
	Seedling mortality	12.97	**0.00**	1.42	2.36	
	Seedling density	13.70	6.99	**0.00**	1.56	
	Seedling aboveground dry mass	79.24	85.34	**0.00**	1.67	Yes
		103.35	105.58	25.66	27.27	No
	Sapling survival	**0.00**	1.00	1.91	0.21	
	Sapling root–shoot dry mass ratio	9.44	2.47	1.68	3.54	Yes
		8.02	0.87	**0.00**	1.84	No
	Sapling total dry mass	3.79	11.54	1.38	1.28	Yes
		2.20	9.93	**0.00**	0.06	No

*Notes*

Bold letters indicate models with ΔAIC < 2, or when more than one model has ΔAIC < 2, bold letters indicate the model with the lowest ΔAIC, indicating the most parsimonious model. ^a^Interaction: considers that the effect of one predictor on the response variable is different at different levels of the other predictor, that is, the influence of seed provenance is different if seeds are in a site or another. ^b^Seed mass inclusion: inclusion (yes) or not (no) of seed mass in the model depending on the significant effect of this covariable in the response variable (adaptive trait).

**Figure 3:**
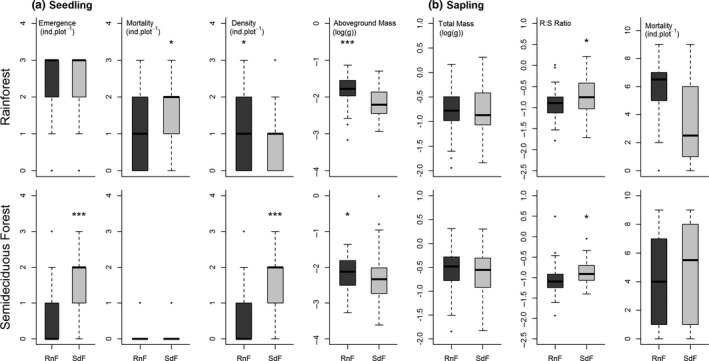
Paired comparisons of the influence of seed provenance (RnF: Rainforest; SdF: Semideciduous Forest) on adaptive traits in reciprocal transplants established with *Euterpe edulis* within these forest types in the Brazilian Atlantic Forest. Mann–Whitney test was used for counting data and *t* test for the log‐transformed data. *p < 0.05; **p < 0.01; ***p < 0.0001

When the plant performance of Rainforest and Semideciduous Forest provenances was compared in their respective sites of origin, we found stronger evidence for local adaptation for seedling establishment, as the influence of seed provenance differed among sites (Table 4). Overall, the local provenances showed the highest plant performance (Figure 3). In the Rainforest site, the local provenance showed higher seedling survival, density, and aboveground dry mass, while in the Semideciduous Forest site, the local provenance showed higher seedling emergence and density (Figure 3). Sapling survival was influenced by seed provenance, site of transplant, and both factors and their interaction, but more markedly by seed provenance, and was highest for the Rainforest provenance in its site of origin (Table 4, Figure 3). Sapling root–shoot dry mass ratio was influenced by seed provenance and site of transplant (Table 4), but did not differ between seed provenances in both sites of transplant (Figure 3). Sapling total dry mass was the only trait showing a strong effect of site of transplant (i.e., higher importance of phenotypic plasticity), with negligible effects of seed provenance (Figure 3). Overall, seedling emergence was favored in the Rainforest, but seedling mortality was also highest in this transplant site (Figure 3).

**Figure 4:**
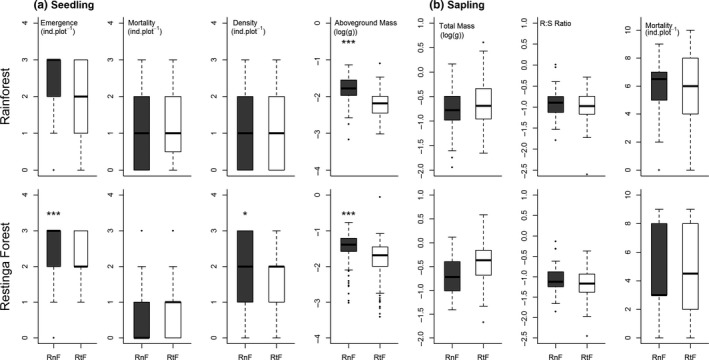
Paired comparisons of the influence of seed provenance (RnF: Rainforest; RtF: *Restinga* Forest) on adaptive traits in reciprocal transplants established with *Euterpe edulis* within these forest types in the Brazilian Atlantic Forest. Mann–Whitney test was used for counting data and t‐test for the log‐transformed data. *p < 0.05; **p < 0.01; ***p < 0.0001

When the Rainforest and *Restinga* forest populations were compared, none of the selected models included the interaction between seed provenance and site of transplant, and for only one trait (seedling mortality), site of transplant was selected for the model. Seed provenance and its combination with site of transplant were selected by most models (Table 4). Seedling mass was higher for the Rainforest provenance compared to the *Restinga* forest in its site of origin, together with seedling emergence and density (Figure 4).

## 4 DISCUSSION

As expected, the more biogeographically divergent ecosystems (i.e., Rainforest and Semideciduous Forest) showed marked neutral and adaptive molecular divergences of populations and substantial evidence of local adaptations. On the other hand, the more closely associated ecosystems (i.e., Rainforest and *Restinga* Forest) showed reduced molecular differentiation and no evidence of local adaptation (or genotypic differentiation). The relative contribution of phenotypic plasticity and local adaptations in favoring range expansion of *E. edulis* therefore depended on the biogeographic context.

In the Semideciduous Forest, the potential local adaptations were related to seedling emergence, which resulted in higher seedling density in the absence of differential mortality and higher root–shoot dry mass ratio for saplings. *E. edulis* seeds are recalcitrant and highly sensitive to desiccation (Brancalion et al., 2011). Seed dispersal occurs in the beginning of the dry season in Semideciduous Forests, while no water limitation for germination exists in Rainforests. Consequently, the higher selective pressure in response to desiccation may have fostered the evolution of ecotypes in Semideciduous Forests with improved germination performance in drier soils. Higher root–shoot dry mass ratio for saplings may also be assumed as an advantage for drier soils, as the preferential allocation of biomass to roots may make seedlings more resistant to drought. Reduced root–shoot dry mass ratio may enhance initial growth and the competitive potential of seedlings in the Atlantic Rainforest, where we expect that light limitation—and not water—is the major driver of seedling mortality. Roots of temperate grassland species can change dramatically in weight and form in order for shoot growth rates to remain reasonably constant (Padilla et al., 2013), and different root–shoot ratios were found for woody and herbaceous species growing in contrasting conditions of water and light availability in Mediterranean‐type ecosystems (Chaves et al., 2002). However, the differential expression of root–shoot ratio by palm provenances in different tropical forest conditions remains unclear.

Seedlings of the Rainforest provenance showed higher aboveground mass in all sites of transplants, which is an indication that early seedling growth can be a major selective filter in humid and shaded forests. In addition, we observed reduced seedling mortality for the local provenance in the Rainforest site when compared to Semideciduous Forest seedlings, which can be related to differential shade tolerance. We observed in the field that seedlings from the Semideciduous population were more subjected to damping off than seedlings of the local provenance. Soil pathogens exert a substantial impact on tree seedling survival (Bertacchi et al., 2016; Mangan et al., 2010), so differential tolerance to pathogens may also be related to local adaptations (Thrall, Burdon, & Bever, 2002). The aforementioned speculations were presented here to contextualize the results of reciprocal transplants in relation to the regeneration conditions of the ecosystems we studied, but need to be experimentally tested to infer the mechanistic ecological processes driving the observed results.

In contrast to the genetic differentiation observed between Semideciduous Forest and Rainforest populations, we did not find clear evidence for differentiation between Rainforest and *Restinga* Forest populations. We are aware that the number of SNPs analyzed is limited to make claims about the genetic targets of selection, but this number was sufficient to distinguish among studied populations in terms of their adaptive divergences. A greater number of genotypes are being analyzed to confirm the results obtained here, as suggested in the studies of Fraser, McGaughran, and Chuah (2016). The Rainforest provenance showed the best performance for most traits evaluated in Rainforest and *Restinga* sites, which can be the result of an inherently reduced vigor of *Restinga* Forest populations (Figure 1). As *Restinga* Forest gene pools can be considered a subset of the gene pool of neighboring Rainforests, founder effects could have led to stronger genetic drift and to inbreeding depression. However, we did not find any evidence of inbreeding depression in the molecular analysis, so the explanation for the reduced performance of *Restinga* Forest seedlings and saplings remains unclear. Adult individuals of *E. edulis* growing in the studied *Restinga* forest are much smaller than those growing in the Submontane Rainforest studied, and this difference was, so far, solely attributed to differential soil nutrient content and salinity (Brancalion et al., 2012).

The use of potentially adaptive SNPs allowed the discrimination of populations according to their potential adaptive divergences, as did neutral microsatellite markers and reciprocal transplants, so the use of this novel approach of genetic analysis may not necessarily yield novel findings in evolutionary studies. Although we have identified the cellular component and molecular function associated with the regions of the genome under selection, we could not associate this information with any specific adaptive traits. On the other hand, linking the supposed cellular and molecular functions to adaptive traits measured in reciprocal transplants and common garden experiments may pave the way for novel findings on the study of local adaptations in plant populations.

The common garden experiment was an ineffective approach for this species (but see Brancalion et al. (2015) for the successful use of common garden for another Atlantic Forest palm). Apparently, differential trait performance requires some level of environmental stimuli to be expressed in common garden experiments, which could be produced by under varying resource availability (Rutherford, Bonser, Wilson, & Rossetto, 2017). Although the evaluation of adaptive traits related to seedling establishment may expand the use of reciprocal transplants in tree species, it would be still fairly unpractical to use this approach for a high number of species. In this case, SNP analysis can provide a valuable tool for large‐scale screenings on adaptive divergence (De Kort et al., 2014).

## CONFLICT OF INTEREST

None declared.

## AUTHOR CONTRIBUTIONS

PHSB, GCXO, and RRR designed the common garden and reciprocal transplant experiments; PHSB implemented and evaluated these experiments; PHSB, MIZ, and MN collected genetic material and performed molecular analysis; SSZ and JM provided support on statistical analysis; RLC provided critical support on palm ecology and evolution; PHSB wrote a first version of the manuscript, and all authors helped to improve it through subsequent reviews.

## DATA ARCHIVING STATEMENT

We intend to archive our data on Zenodo.
