# Supplementary figures and images for: Phenotypic plasticity and local adaptation favor range expansion of a Neotropical palm
Source: Ecol Evol. 2018 Jul 3;8(15):7462–75. doi: 10.1002/ece3.4248 (PMC6106193; doi:10.1002/ece3.4248)

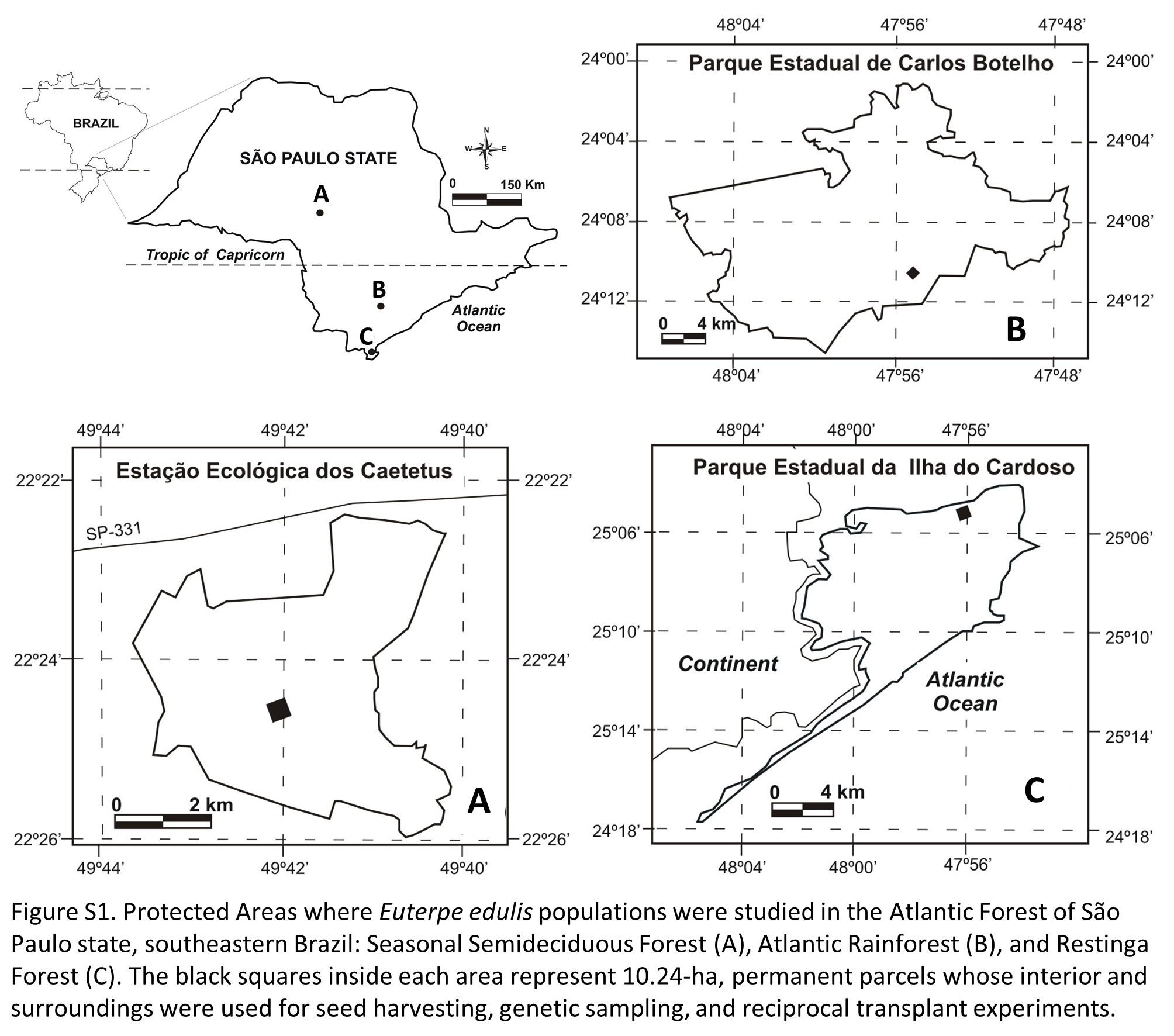

Supplement: Supplementary file 1 [file ECE3-8-7462-s001.tif]

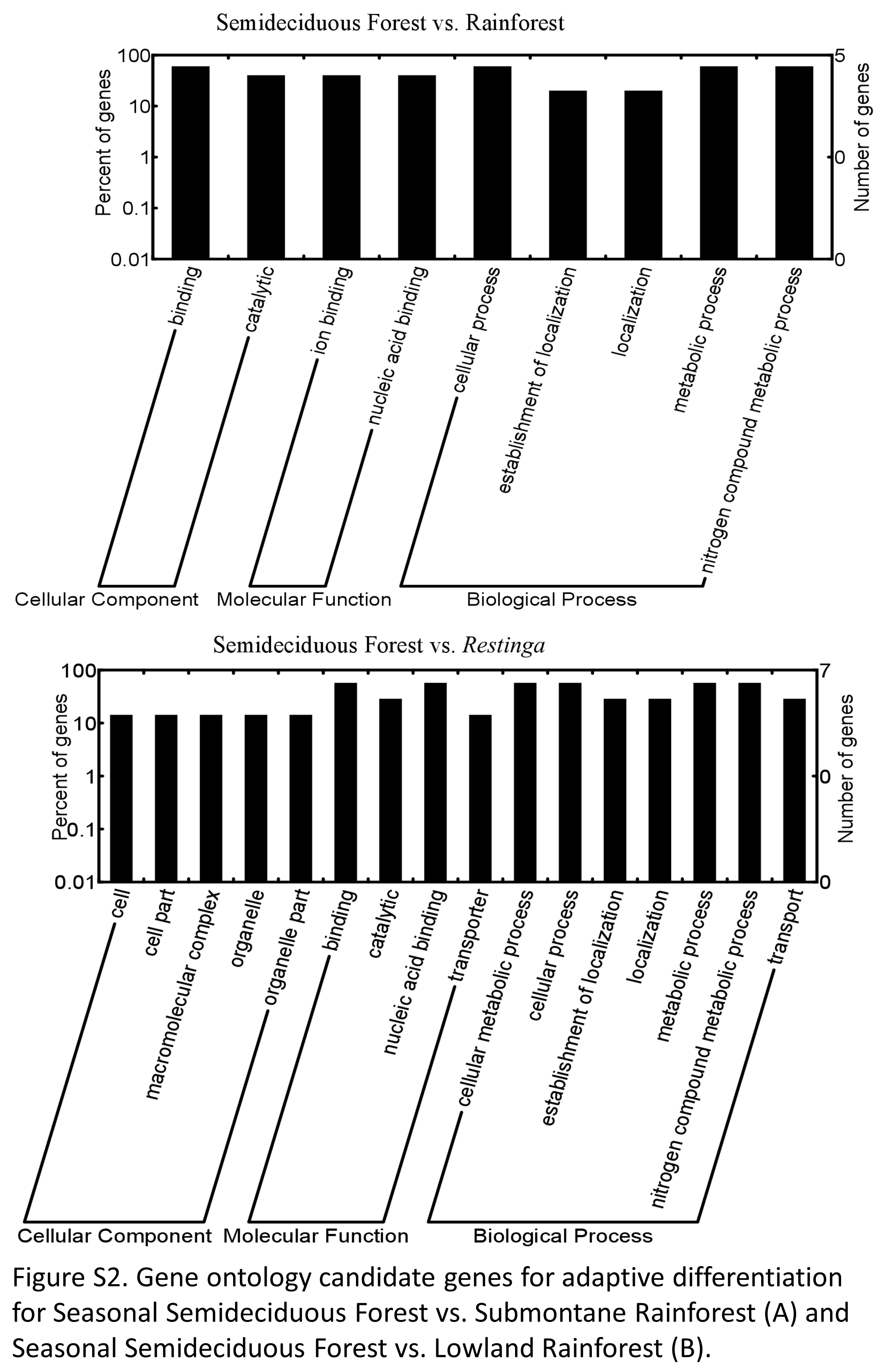

Supplement: Supplementary file 2 [file ECE3-8-7462-s002.tif]
