# Supplementary material for: Phenotypic plasticity and local adaptation favor range expansion of a Neotropical palm
Source: Ecol Evol. 2018 Jul 3;8(15):7462–75. doi: 10.1002/ece3.4248 (PMC6106193; doi:10.1002/ece3.4248)
Supplement: Supplementary file 3 [file ECE3-8-7462-s003.docx]

Table S1: Environmental characteristics of the sites were *Euterpe edulis* populations were studied in Atlantic Forest of Brazil.

| Biotic and abiotic characteristics | Forest types | | |
| --- | --- | --- | --- |
|  | Restinga Forest | Semideciduous Forest | Rainforest |
| Climate (Köepen classification) | Equatorial climate (Af), with strong influence of oceanity, mean temperature = 22.4ºC and annual rainfall = 2261 mm, without dry season. | Humid sub-tropical climate (Cwa), with dry season in the winter (water deficit = 10 mm), mean temperature = 21.4°C and annual rainfall = 1303 mm. | Equatorial climate (Af), mean temperature = 21.8ºC and annual rainfall = 1582 mm, without dry season. |
| Topography | Coastal-flat, next the sea level (average altitude = 7 m) | Flat to slightly mountainous (average altitude = 522 m) | Mountainous (average altitude = 400 m) |
| Predominant soil | Ferrocarbic Spodosol: hydromorfic, sandy (90% sand, 4% silt, 6% clay), low nutrient status (P = 3.3 mg/kg, K = 0.8 mmol/kg, Ca = 1.9 mmol/kg, Mg = 3.0 mmol/kg), low pH (pH_CaCl2_ = 3.0) and high aluminum content (Al^3+^ = 9.4 mmol/kg) | Ultisol: well-drained, sandy clay loam (78% sand, 10% silt, 12% clay), high nutient status (P = 4.9 mg/kg, K = 3.1 mmol/kg, Ca = 38.7 mmol/kg, Mg = 8.5 mmol/kg) and moderate pH (pH_CaCl2_ = 5.1) and almost nule aluminum content | Cambisol: clay (45% sand, 17% silt, 38% clay), low nutient status (P = 3.5 mg/kg, K = 1.9 mmol/kg, Ca = 3.8 mmol/kg, Mg = 3.2 mmol/kg) and low pH (pH_CaCl2_ = 3.8) and high aluminum content (Al^3+^ = 20 mmol/kg) |
| Floristic richness of a 10ha Permanent Plot established in the site (diameter at breast height - dbh > 4.8 cm) | 114 species and 43 families | 148 species and 44 families | 205 species and 44 families |
| *Euterpe edulis* abundance (dbh > 4.8 cm) | 19.8% of total individuals (1^th^ most abundant species - 300 individuals/ha) | 0.1% of total individuals (65^th^ most abundant species - 1.3 individuals/ha) | 21.5% of total individuals (1^th^ most abundant species - 240 individuals/ha) |
| Dbh (> 4.8 cm, *X* ± DP) of *Euterpe edulis* individuals | 7.4 ± 2 cm, *N* = 3,076 | 9.92 ± 3 cm, *N* = 100 | 10.3 ± 4 cm, *N* = 2,307 |
