# Supplementary material for: Phenotypic plasticity and local adaptation favor range expansion of a Neotropical palm
Source: Ecol Evol. 2018 Jul 3;8(15):7462–75. doi: 10.1002/ece3.4248 (PMC6106193; doi:10.1002/ece3.4248)
Supplement: Supplementary file 4 [file ECE3-8-7462-s004.docx]

Table S2. Characterization of gene ontology annotations obtained in blast2go analysis for *Euterpe edulis* populations growing in three forest types Semideciduous Forest – SdF; Rainforest – RnF; and *Restinga* Forest – RtF within the Brazilian Atlantic Forest.

| **Outliers** | **Description** | **#Hits** | **e-Value** | **mean similarity** | **#gene ontology** | **SdF vs. RnF** | **SdF vs. RtF** | **RnF vs. RtF** | **Gene ontology names list** |  |  |
| --- | --- | --- | --- | --- | --- | --- | --- | --- | --- | --- | --- |
| 43 | gag-pol poly | 20 | 1.91E-6 | 76.3% | 4 | x | x |  | P:RNA phosphodiester bond hydrolysis, endonucleolytic; F:nucleic acid binding; F:RNA-DNA hybrid ribonuclease activity; P:DNA integration |  |  |
| 31 | Copia | 20 | 5.65E-12 | 90% |  | x | x |  |  |  |  |
| 57 | PREDICTED: uncharacterized protein At2g24330 | 2 | 5.46E-4 | 73% |  | x | x |  |  |  |  |
| 77 | retrotransposon Ty1-copia subclass | 20 | 5.96E-10 | 92.8% | 4 | x | x |  | F:DNA binding; F:zinc ion binding; P:DNA recombination; P:DNA integration |  |  |
| 7 | histidine phosphotransferase | 20 | 7.57E-12 | 87.7% | 1 | x | x | x | F:transferase activity |  |  |
| 14 | retroelement pol poly | 20 | 2,00E-08 | 76.35% | 2 | x | x |  | P:nucleic acid metabolic process; P:cellular macromolecule metabolic process |  |  |
| 20 | rve domain-containing RVT_3 domain-containing | 20 | 6.41E-7 | 78.75% |  | x | x |  |  |  |  |
| 22 | pentatricopeptide repeat-containing mitochondrial-like | 7 | 2.18E-5 | 85% |  | x | x |  |  |  |  |
| 27 | Heavy metal-associated isoprenylated plant 26 | 20 | 1.6E-13 | 96.65% | 2 | x | x |  | F:metal ion binding; P:metal ion transport |  |  |
| 28 | hypothetical protein F511_43985 | 17 | 2.05E-4 | 75% |  | x | x |  |  |  |  |
| 87 | ATP synthase CF0 subunit IV chloroplast | 20 | 4.67E-12 | 95.2% | 6 |  | x |  | C:proton-transporting ATP synthase complex, coupling factor F o ; C:integral component of membrane; C:plasma membrane; P:ATP synthesis coupled proton transport; |  |  |
| 91 | retrotransposon Ty1-copia subclass | 20 | 3.94E-6 | 98.05% | 6 |  | x |  | F:nucleic acid binding; F:DNA binding; F:zinc ion binding; F:metal ion binding; P:DNA integration; P:DNA recombination |  |  |
